# Microleakage of Single-Cone Gutta-Percha Obturation Technique in Combination with Different Types of Sealers

**DOI:** 10.7508/iej.2015.03.011

**Published:** 2015-07-01

**Authors:** Saeedeh Sadr, Ali Golmoradizadeh, Maryam Raoof, Mohammad Javad Tabanfar

**Affiliations:** a *Persian Gulf Oral and Dental Diseases Research Center, Hormozgan University of Medical Sciences, Bandar Abbas, Iran; *; b *Laboratory of Molecular Neuroscience, Neuroscience Research Center, Institute of Neuropharmacology, Kerman University of Medical Sciences, Kerman, Iran; *; c *Department of Endodontics, Dental School, Kerman University of Medical Sciences, Kerman, Iran; *; d *Student of Research Committee, Hormozgan University of Medical Sciences, Bandar Abbas, Iran*

**Keywords:** Dye Penetration, Microleakage, Obturation, Root Canal Therapy, Sealer, Single-Cone

## Abstract

**Introduction:**

Various materials and methods have been recommended for successful root canal obturation. The aim of this experimental *in vitro* study was to compare the sealing ability of three root canal sealers AH-26, glass ionomer cement (GIC) and zinc oxide eugenol (ZOE) in single gutta-percha obturating system.

**Methods and Materials:**

Seventy extracted single-rooted human teeth were decoronated. The teeth were randomly divided into 3 experimental groups (*n*=20) and 2 positive and negative control groups. After root canal preparation, canals were obturated with single-cone method using either AH-26, GIC and ZOE. The leakage was evaluated using the dye penetration method. The samples were sectioned to evaluate the linear leakage using a stereomicroscope. The data were analyzed using the One-way ANOVA test.

**Results:**

All the specimens in the positive control group showed evidence of leakage. In the experimental groups, the lowest leakage scores were observed in the AH-26 group (*P*<0.05). However, there were no statistically significant differences between GIC and ZOE samples (*P*=0.676).

**Conclusion:**

AH-26 showed a superior seal and less microleakage compared to the two other materials in single gutta-percha obturating system.

## Introduction

The success of a root canal therapy strongly depends on creating a fluid-tight apical and coronal seal [1-5]. Various materials and methods have been introduced for obturating an instrumented root canal [6]. Endodontic sealers play a critical role in providing an impervious seal. They fill the irregularities and minor discrepancies between the root canal walls and core filling material [7-10]. However, inappropriate sealer coating may result in voids and permit bacterial microleakage which can potentially lead to treatment failure [11, 12]. A variety of sealers have been used for this purpose including zinc oxide-eugenol (ZOE)-based cements, glass ionomer cements, polymer-based sealers, calcium hydroxide-based sealers and silicon-based sealers [6].

The popularity of single-cone obturation technique is increasing because of widespread using of rotary nickel-titanium (NiTi) instruments and matched-taper gutta-percha cones. Moreover, this technique is considered simple, improves practice and causes less stress for both patient and to clinician [5, 13, 14]. In one study, there was no significant difference between different obturation methods including single-cone techniques, lateral and vertical condensation of gutta-percha, Thermafil, and Ultrafil techniques [14].

Several studies evaluated the apical microleakage of the single-cone technique [15-20]. Damasceno *et al.* [15] assessed the apical microleakage of the single-cone technique of the ProTaper system compared with thermoplasticized TC (thermometer-controlled heating) obturation (Tanaka de Castro & Minatel Ltda., Cascavel, PR, Brazil) system without master cone and together with AH-Plus sealer. The results showed apical microleakage in both techniques; however, statistically significant differences were not detected. Holland *et al.* [16] evaluated the effect of sealer type and filling technique on the apical marginal microleakage, using the single-cone and lateral condensation methods. The authors reported that the single-cone technique showed less marginal leakage than lateral condensation, but it was characterized by overfilling in all cases, which did not occur with the lateral condensation technique. Wu *et al.* [19] estimated the long-term apical leakage of the single-cone technique in teeth filled with RoekoSeal cement. The authors concluded that in long and straight canals, the single-cone technique prevented the fluid infiltration after one year. Yilmaz *et al.* [20] compared the apical efficacy of the BeeFill 2in1 (VDW, Münich, Germany), System B heating device (Analytic Technology, Redmond, WA, USA) and Obtura II systems (Spartan/Obtura, Fenton, Missouri, USA) with the single-cone and cold lateral compaction techniques at one and two weeks. There were no differences in the apical seal of the root canals filled with either of the techniques; however, they were not capable of completely blocking the fluid conductance. Monticelli *et al.* [21] compared the apical sealing of two systems of single-cone obturation Activ GP/glass ionomer sealer (Brasseler USA, Savannah, GA) and GuttaFlow (Coltène/Whaledent Inc, Cuyahoga Falls, OH, USA) with the vertical condensation technique and AH Plus cement by using a model of bacterial infiltration in single-rooted teeth. The authors concluded that both single-cone techniques did not promote a durable apical sealing.

The aim of the present *in vitro* study was to compare the coronal and apical seal of canals obturated with different sealers including AH-26, glass ionomer cement (GIC) and zinc oxide eugenol (ZOE) in single gutta-percha obturating system.

## Materials and Methods

Seventy newly extracted human anterior single-rooted teeth were selected for this study. Radiographs and visual inspection under a stereomicroscope at 20× magnification (Olympus BX50, Japan) were used to verify any open apices, cracks, resorptive defects and canal calcifications. The teeth were immersed in 5.25% NaOCl solution for 5 min. Subsequently, the samples were cleaned of tissue remnants and calculus and then rinsed and stored in normal saline. The crowns of the teeth were decoronated using a high speed handpiece under continuous water spray. All the procedures were performed by a single operator. Working length (WL) was determined by inserting a K-file# 15 (Mani, Nakanishi Inc., Tokyo, Japan) into the canal until it was just visible at the apical foramen at 10× magnification; then 1 mm was subtracted from this measurement. The root canals were prepared using ProTaper rotary instruments (Dentsply Maillefer, Ballaigues, Switzerland) installed on an electrical endodontic handpiece (Endo Mate DT, NSK, Japan) at speed and torque of 250 rpm and 300 Ncm, respectively. Preparation was carried out according to the manufacturer’s recommendations using the crown-down technique. Briefly, the S1 file was used to clean and shape the coronal part of the canal. Subsequently, the SX file was used to increase the taper of the coronal region and S1, S2, F1, F2 and F3 were used sequentially to the full WL. A new set of instruments was used for each group of teeth. No instrument fracture occurred during preparation of the specimens. The canals were irrigated between instruments with 10 mL of freshly prepared solution of 5.25 % NaOCl carried up to the apical 3 mm with 27-gauge disposable plastic syringes needle tips placed passively into the canal. Following instrumentation, root canals were irrigated with 1 mL EDTA 17% (Asia Chemi Teb Co., Tehran, Iran) followed by 5 mL 2.5% NaOCl to remove the smear layer. Finally, the root canals were flushed with 3 mL of saline solution and dried with paper points.

The teeth were randomly divided into 5 groups, consisting of three experimental groups (*n*=20) and two negative and positive control groups (*n*=5).

In all of the groups, root canal obturation was carried out using the single-cone obturation technique. The sealers were carried into the canals using a lentulo spiral (Mani, Tochigi, Japan). In group 1, AH-26 sealer (Dentsply, DE Trey, Konstanz, Germany); in group 2, glass ionomer cement (GIC) type I (GC Corporation, Tokyo, Japan) and in group 3, zinc oxide eugenol (ZOE) cement (Zinc Oxide 99.86%, Golchadent, Iran) were used. An F3 ProTaper gutta-percha cone coated with the sealer was used as a master cone and was inserted to the canal space up to the WL. The excess gutta-percha was removed with a heat carrier and the remaining gutta-percha was vertically compacted at the canal orifice. The access cavities were sealed with Cavit (ESPE-Premier, Norristown, PA, USA). 

In the positive control samples, the teeth were obturated with single ProTaper gutta-percha cone without sealer. Samples in the negative control group had the entire root sealed with sticky wax (Kerr, Romulus, MI, USA). All samples were incubated for 1 week at 37^°^C and 100% humidity to allow complete setting of the sealers. In all the specimens, except for the positive controls, external root surfaces were covered by two layers of three different colors of nail varnishes, excluding the coronal and apical 1 mm of the roots. However, the root surfaces of the negative control teeth were entirely coated with two layers of nail varnish. The teeth were then placed into 5% methylene blue dye solution for seven days at 37^º^C. After one week the samples were removed from the dye solution and the roots were rinsed for 15 min under tap water and dried. The nail varnish was removed with a scalpel. The samples were sectioned longitudinally in a bucco-lingual direction from coronal to apical. For each sample, dye penetration was measured in millimeters under stereomicroscope (Olympus BX50, Japan) at 40× magnification. The results were analyzed using the one-way analysis of variance (ANOVA). The level of significance was set at 0.05.

**Figure 1:**
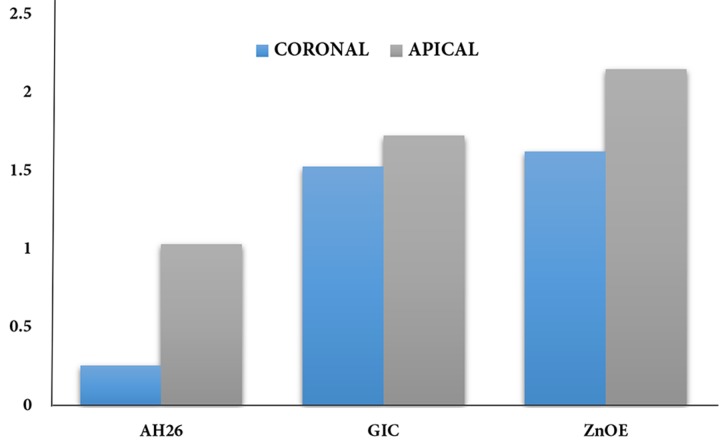
Mean (SD) of dye penetration in different groups

## Results

The positive control teeth showed maximum dye penetration and leaked at least 5 mm into the canals. However, no leakage was observed in the negative control group. 

The mean±SD values of microleakage are demonstrated in Table 1 and Figure 1. AH-26 group had the least amount of microleakage compared to the other groups (*P*<0.05). 

The difference in coronal and apical leakage between AH-26 and other groups was significant (*P*<0.05). Also the difference between GIC and ZOE groups was statistically significant in terms of the apical leakage (*P*=0.018). Moreover, there were no statistically significant differences in coronal leakage between GIC and ZOE groups (*P*=0.676).

## Discussion

The present study compared the sealing ability of different sealers in single-cone obturation method. Although dye penetration was observed to different degrees in all of the experimental samples, AH-26 demonstrated the least amount of microleakage. 

The results may be related to the considerable bond strength of AH-26 to dentin as well as gutta-percha. Consistent with our results, Lee *et al.* [22] compared the bond strength of Kerr Sealer, SealApex, Ketac-Endo and AH-26, to dentin and gutta-percha cones. They reported higher bond strength values for AH-26. Tagger *et al.* [23] also found that AH-26 had a significantly superior bond to gutta-percha than a ZOE-based sealer. De Gee *et al.* [24] conducted a study on the sealing capacity of AH-26 or GI-based sealer (Ketac-Endo); when the sealers were used in bulk between two opposing dentine surfaces, the leakage of Ketac-Endo samples was more than AH-26. After shear loading, it was found that the area of adhesive failure was 88% and 15% for Ketac-Endo and AH-26, respectively. In contrast, Pommel *et al.* [25] found no statistically significant differences between AH-26 and Ketac-Endo regarding the apical leakage. Discrepancies between the results may stem from the differences in the methodology used for microleakage evaluation. Clinical data is required to provide further evidence to support either argument.

For many years, ZOE-containing sealers including Roth’s 811, Kerr EWT, Rickert’s sealer, Procosol and Wach’s sealer have been the most popular and widely used sealers [26]. On the other hand, AH-26 is an epoxy resin-based sealer that was initially developed as a single obturation material. Because of its positive handling characteristics, good flow, adherence to dentin walls and sufficient working time, AH-26 has been extensively used as a sealer [27]. Moreover, in many studies, GI cements have been used as comparative sealers [26]. Due to the above points, we used these three types of sealers for our study.

Various studies have employed different methods to evaluate apical and coronal microleakage like the degree of dye penetration, radioisotope penetration, bacterial penetration, electrochemical means and fluid filtration techniques. However, no concrete results are available that prove the superiority of one sealer over the others. Dye penetration method is a common technique for microleakage studies [28-32]; the advantages are low cost, low toxicity, good availability and ease of storage [28]. Torabinejad *et al.* [31] has stated that if a root filling material does not allow penetration of small particles such as dye molecules, it is more likely to have the potential to prevent microleakage of bacteria and their by-products. As methylene blue has a low molecular weight and penetrates more deeply along the root canal filling [33], we used it as a leakage marker for the current study.

As Van der Sluis *et al.* [34] showed significant differences in leakage between oval and round canals, we selected single-rooted teeth with straight and round canals for our study.

It is well known that root filling materials penetrate better into dentinal tubules in the absence of the smear layer [32]. In the present investigation, 17% EDTA and 5.25% NaOCl were used as materials to remove the smear layer [35]. 

Among the different materials/techniques introduced for root obturation, cold laterally compaction of gutta-percha in combination with a sealer is the most widely accepted and used obturation technique. However, many studies have shown that this approach fails to provide a fluid-tight seal of the root canal system, due to incorporation of apical voids, the lack of surface adaptation, and resorption of the sealer component with time. Some attempts have been made to resolve this problem through variations in obturation techniques. Among these, single-cone filling of root canals has been introduced to minimize the sealer component through the gutta-percha cones that closely match the geometry of nickel–titanium instrumentation systems [36]. These cones ensure 3-dimensional obturation of the root canal over its entire length without necessitating accessory cones or the time spent on lateral condensation [14]. Single-cone gutta-percha obturation is not only rejected, but also is becoming more popular because of simplicity and time saving [14]. Studies have shown controversial results of the efficacy of this method. [17, 20, 37]. So, more research in this field can be beneficial to have more information.

**Table 1:** Mean (SD) and minimum/maximum of microleakage in different experimental groups [confidence interval (CI)=95%]

**Sealer (N)**	**Mean (SD)**	**Minimum**	**Maximum**
**AH-26 (20)**	0.25 (0.23)	0.00	0.96
**GI (20)**	1.52 (0.26)	1.12	2.23
**ZOE (20)**	1.62 (0.49)	0.76	2.85
**Total (60)**	1.13 (0.71)	0.00	2.85

## Conclusions

Within the limitation of the present study, AH-26 sealer had better apical and coronal sealing ability than GIC and ZOE sealers in single gutta-percha obturating system. However, further long-term studies are necessary to establish the clinical performance of single gutta-percha obturating system in conjunction with different sealers.
